# Determining chemical air equivalency using silicone personal monitors

**DOI:** 10.1038/s41370-021-00332-6

**Published:** 2021-05-05

**Authors:** Steven G. O’Connell, Kim A. Anderson, Marc I. Epstein

**Affiliations:** 1 MyExposome, Inc., Corvallis, OR USA; 2 Environmental and Molecular Toxicology Department, Oregon State University, Corvallis, OR USA

## Abstract

**Background:**

Silicone personal samplers are increasingly being used to measure chemical exposures, but many of these studies do not attempt to calculate environmental concentrations.

**Objective:**

Using measurements of silicone wristband uptake of organic chemicals from atmospheric exposure, create log *K*_*sa*_ and *k*_*e*_ predictive models based on empirical data to help develop air equivalency calculations for both volatile and semi-volatile organic compounds.

**Methods:**

An atmospheric vapor generator and a custom exposure chamber were used to measure the uptake of organic chemicals into silicone wristbands under simulated indoor conditions. Log *K*_*sa*_ models were evaluated using repeated k-fold cross-validation. Air equivalency was compared between best-performing models.

**Results:**

Log *K*_*sa*_ and log *k*_*e*_ estimates calculated from uptake data were used to build predictive models from boiling point (BP) and other parameters (all models: *R*^2^ = 0.70–0.94). The log *K*_*sa*_ models were combined with published data and refined to create comprehensive and effective predictive models (*R*^2^: 0.95–0.97). Final estimates of air equivalency using novel BP models correlated well over an example dataset (Spearman *r* = 0.984) across 5-orders of magnitude (<0.05 to >5000 ng/L).

**Significance:**

Data from silicone samplers can be translated into air equivalent concentrations that better characterize environmental concentrations associated with personal exposures and allow direct comparisons to regulatory levels.

## Introduction

Using silicone wristbands (SWBs) to measure personal chemical exposures is an approach less than 7 years old [1], but is becoming more widely used in the last 2 years [2–14]. SWBs have been found to have high compliance even among children [15, 16], and have been found to correlate well with internal biomarkers of exposure from urine and blood sampling [17–19]. Most research endeavors using SWBs measure volatile organic compounds (VOCs) or semi-volatile organic compounds (SVOCs) and analyze results from the passive sampling extracts without estimating environmental concentrations during exposure. While there are multiple routes of exposure that SWBs may capture [1, 5, 20], even attempting to estimate air concentrations are often left out of current research. Models and equations used to estimate environmental concentrations from passive sampling can be challenging to use in practice, which then leaves many researchers more inclined to rely on extract concentration data exclusively.

While still effective, there are limitations with only comparing extract concentrations since uptake into the silicone is not normalized between compounds. Typically extract concentrations are given in ng/g silicone/day or pmol/SWB. Comparisons of extract data are appropriate when comparing individual chemicals among samples within the same study but can limit comparisons between chemicals or studies. Specifically, the following problems apply: (1) interpreting relative abundance between chemicals as directly reflective of environmental exposure abundance, (2) reporting environmental forensic ratios that are not normalized for differences in uptake (i.e., at least normalized by log *K*_*sa*_ values); (3) comparisons between studies if data is not normalized consistently, and (4) comparisons of extract data to regulatory levels.

Calculating environmental concentrations from passive sampling devices is well established in theory [21–24], and within the past two decades estimating environmental concentrations using specifically silicone is becoming more common [25–30]. Using established rate constant-based equations derived from Fick’s first law of diffusion for compounds in any phase of uptake [21], calculating environmental concentrations (*C*_*a*_) involves five input parameters. These parameters include: deployment time (*t*), amount of sampler (ex: *V*_*s*_), the amount of compound in the sampler (*N*), a rate-based parameter (*R*_*s*_ or *k*_*e*_), and finally a partition coefficient (ex: *K*_*sa*_) that describes the ratio of passive sampler concentration to environmental concentration at equilibrium under deployment conditions (Eqs. (1) and (2)).

1 $$C_a = \frac{{N_{compound}}}{{V_s \times K_{sa} \times \left( {1 - e^{\left( { - k_e \,\times\, t} \right)}} \right)}}$$

or

2 $$C_a = \frac{{N_{compound}}}{{V_s \times K_{sa} \times \left( {1 - e^{\left( { - \frac{{R_s \,\times\, t}}{{V_s \,\times\, K_{sa}}}} \right)}} \right)}}$$

Environmental estimates are more reliable and accurate if partition coefficients and rate-based parameters are empirically derived or predicted using comprehensive models. For personal silicone samplers and other passive samplers, partition coefficients that describe the ratio of sampler concentration relative to environmental concentration at equilibrium (ex: *K*_*sa*_) are especially critical to the accuracy of the resulting environmental estimate [31, 32]. This is due to *K*_*sa*_ values expressed in log scale as well as its inherent mathematical influence in the environmental concentration equation itself (see Eqs. (1) and (2) as well as Supporting Information). In addition, there are rate-based parameters (e.g., the sampling rate, *R*_*s*_, or the dissipation rate, *k*_*e*_) that are necessary to predict or obtain in order to achieve accurate environmental concentrations.

For researchers using silicone personal samplers, it would be helpful to have comprehensive models with well-established and easy to utilize inputs to help predict *K*_*sa*_ and other uptake parameters for SWBs or other silicone samplers such as brooches, necklace tags, and pet tags [5, 13, 14]. Recent research estimated partition coefficients (*K*_*sa*_) between SWBs and air for SVOC compounds, assuming consistent temperature, humidity, and wind speed [7]. Keeping environmental conditions such as temperature and humidity consistent will limit confounding factors that lead to inaccurate estimates. In addition, adding VOC compounds to existing research efforts will help make resulting models more comprehensive and accurate over the wide breadth of chemistry that can be targeted with silicone. When assuming consistent environmental parameters, it is helpful to use the term “equivalent” to describe the resulting environmental concentrations since the estimates could differ during deployment. While it is possible to estimate sampling rates (*R*_*s*_) or *k*_*e*_ in situ using compounds infused prior to deployment [23], or adjust for environmental conditions mathematically [33], it nonetheless important to establish these parameters under consistent conditions for as many compounds as possible before developing environmental adjustments to parameters like *K*_*sa*_ and *k*_*e*_.

The goals of this study are: (1) to expand upon measurements of atmospheric uptake using SWBs for both VOC and SVOC compounds, (2) to build, test, and compare models of uptake parameters using inputs from established and convenient sources, and (3) to provide an example of calculating air equivalent concentrations using the best available models. To achieve those objectives, we used a custom exposure setup and a gas vapor generator to measure the uptake of chemicals from the atmosphere into SWBs for VOC (*n* = 14) and SVOC (*n* = 8) compounds. Using consistent environmental conditions to represent a standard indoor environment i.e., 25 °C, 50% humidity, nominal wind speed (<0.15 m/s), we were able to calculate uptake parameters like *K*_*sa*_ and *k*_*e*_. From these calculated parameters, models were generated using physicochemical estimates from established databases (i.e., EPA’s Comptox database). Our predictive parameter models were tested using cross-validation (CV) to determine the best performing model and then values were compared with other published models. Finally, air equivalency estimates were compared using sets of the best performing models to exemplify the agreement between calculations and discuss limitations of air equivalency when using SWBs or other silicone personal samplers.

## Methods

### Supplies, materials, and instrumentation

A total of 22 VOC and SVOC chemicals were chosen for passive sampling uptake experiments that represented a wide variety of chemistries (Table 1). Analytical grade standards (purity ≥ 97%), either liquid or in permeation tubes, were obtained from several vendors including AccuStandard, Inc. (New Haven, CT), Kin-tek Analytical (La Marque, TX), and VICI Metronics (Poulsbo, WA). All solvents used were at least Optima-grade (Fisher Scientific, Pittsburg, PA) or equivalent. Water used to rinse silicone or glassware was deionized water (DI) or ultrapure water. Orbital shaker and closed-cell solvent evaporators were obtained from VWR (Radnor, PA), and Biotage (Charlotte, NC), respectively. Air-tight bags used for the storage of SWBs were composed of fluoropolymers (PTFE) and obtained from Welch Fluorocarbon (Dover, NH).

**Table 1:** Chemicals targeted in the experimental setup along with physicochemical data, exposure concentrations, and extraction methods.

Compound	CAS	Abbreviation	VOC/SVOC	MW	BP-Exp (°C)	BP-TEST (°C)	BP-OPERA (°C)	log *K*_*oa*_ OPERA	Vapor pressure —OPERA (mm Hg at 25 C)	Exposure Conc. (ng/L)	Extraction
Dichloromethane	75-09-2	DCM	VOC	85	40	44.4	40.0	2.29	4.33E+02	2470	Liquid–MeOH
Acrolein	107-02-8	ACO	VOC	53	52	52.4	52.7	2.11	2.72E+02	625	Liquid–MeOH
Acrylonitrile	107-13-1	ACY	VOC	53	77	107	77.4	2.44	1.07E+02	4360	Liquid–MeOH
Methyl ethyl ketone	78-93-3	MEK	VOC	72	80	85.9	79.3	2.73	9.06E+01	4540	Liquid–MeOH
Benzene	71-43-2	BEN	VOC	78	80	96.2	80.1	2.83	9.47E+01	1600	Liquid–MeOH
Toluene	108-88-3	TOL	VOC	92	111	128	111	3.49	2.83E+01	3800	Liquid–MeOH
1,1,2-Trichloroethane	79-00-5	112-TCA	VOC	133	114	113	114	3.35	2.35E+01	1790	Liquid–MeOH
4-Methyl-2-pentanone	108-10-1	MIBK	VOC	100	117	119	116	3.58	1.97E+01	4620	Liquid–MeOH
1,1,2,2-Tetrachloroethane	79-34-5	1122-TCA	VOC	168	147	132	147	3.46	4.67E+00	3100	Liquid–MeOH
p-Xylene	106-42-3	XYL	VOC	106	138	141	141	3.84	8.84E+00	166	Thermal
1,2,4-Trimethylbenzene	95-63-6	1,2,4-TMB	VOC	120	169	178	170	4.54	2.10E+00	65.8	Thermal
1,3-Dichlorobenzene	541-73-1	1,3-DCB	VOC	147	173	182	173	4.16	2.15E+00	182	Thermal
Undecane	1120-21-4	UDEC	VOC	156	196	190	196	5.01	4.12E−01	42.7	Thermal
1,2,4-Trichlorobenzene	120-82-1	1,2,4-TCB	VOC	181	214	226	213	4.96	4.59E−01	74.8	Thermal
2,4-Dinitrotoluene	121-14-2	2,4-DNT	SVOC	182	300	305	300	5.08	1.48E−04	4.46 and 3.76	Liquid–EA
Hexachlorobenzene	118-74-1	HCB	SVOC	285	325	293	325	7.37	1.82E−05	117 and 0.590	Liquid–EA
Tris(chloroethyl)phosphate	115-96-8	TCEP	SVOC	285	330	295	330	8.41	6.09E−02	2.61	Liquid–EA
Phenanthrene	85-01-8	PHE	SVOC	178	340	341	340	7.55	1.12E−04	1.50	Liquid–EA
Chlorpyrifos	2921-88-2	CLPYF	SVOC	351	*–*	–	364	10.6	2.03E−05	3.49	Liquid–EA
Pyrene	129-00-0	PYR	SVOC	202	404	415	394	8.86	4.48E−06	0.307	Liquid–EA
Fluoranthene	206-44-0	FLA	SVOC	202	384	378	394	8.86	9.08E−06	0.506	Liquid–EA
Permethrin	52645-53-1	PERN	SVOC	391	*–*	412	401	11.7	2.19E−08	0.162	Liquid–EA

*BP Exp* boiling point was obtained from EPISuite software database citing experimental data

Other physicochemical estimates (BP, log *K*_*oa*_, vapor pressure) were obtained from EPA Comptox database QSARs (quantitative structure-activity relationships) [37], *TEST* toxicity estimation software tool [35], *OPERA* open structure-activity/property relationship app [36].

### SWB pre-treatment

Prior to use as a passive sampler, commercially purchased SWBs (24hourwristbands.com, regular size, ~4.76 g) are rinsed with water to remove dust and particles from manufacturing, and heated in a vacuum oven up to at least 270 °C to remove impurities as described previously [34]. The rinsed and conditioned wristbands cool in the oven under vacuum and are then immediately stored in air-tight containers. Along with each batch of conditioned SWBs, samplers were extracted and analyzed to ensure batch-to-batch conditioning met quality objectives as described previously [1].

### Kinetic study design and setup

**Fig. 1:**
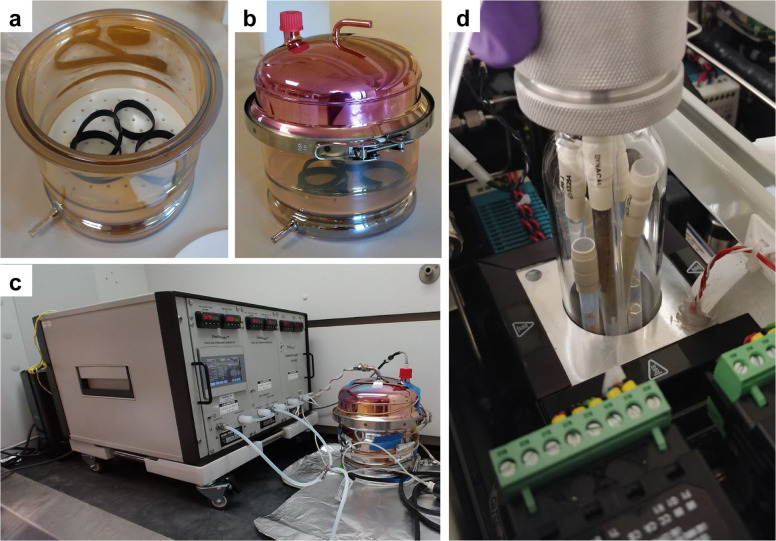
Experimental setup. **a** Each time series consisted of 5–6 SWBs, with one taken out at a designated time point during the study period. **b** The exposure chamber had a coating applied to the glass to prevent unwanted adherence of chemicals to the chamber during testing. **c** The Kin-tek vapor generator was supplied with nitrogen and connected to the exposure chamber with PTFE tubing, the chamber was wrapped in a heating cord to ensure temperature stability, and the entire setup was placed inside a fume hood for safety. **d** Multiple permeation tubes containing one chemical per tube were placed inside the permeation chamber and heated to pre-certified temperatures within 0.1 °C.

This study included 3–14-day experiments and used a Kin-tek vapor generator (FlexStream SD, with humidification and dilution modules, Houston, TX) with certified permeation tubes to provide known chemical atmospheric exposure to SWBs from ppt to ppm levels (Fig. 1). Paired with the Kin-tek system was a custom-built glass exposure chamber (~7 L volume, 20 cm inner diameter, Adams & Chittenden, CA) capable of holding all WBs for each time series (*n* = 5–6) and provides disruptive flow to homogenize exposures within the chamber (Fig. 1a). Within the vapor generator, all lines and valves (PTFE—polytetrafluoroethylene, glass, or stainless steel) were heated to reduce unwanted adherence of compounds before exiting the instrument, and all glass surfaces of the exposure chamber were coated by SilcoTek® to reduce adherence of compounds within the chamber (Fig. 1b). Purified gas (N_2_, >99.9% purity) was used to perform all kinetic experiments with gas delivery ranging 951–6391 mL/min prior to entering the large chamber. Equipment including heating cords (Briskheat, OH) and temperature data loggers (Madgetech, NH) were tested prior to use, and data including flow rate, calculated air concentration for each chemical, humidity, and temperature were recorded every 1–30 min depending on application and study duration using manufacturer software. All experimentation took place within a fume hood and the exhaust was directed to the back of the fume hood for safety precautions and to help maintain an open system of exposure (Fig. 1c).

The temperature and humidity during exposures were designed to resemble realistic and standardized “indoor” conditions, which were set at 25 °C and 50% relative humidity (RH) respectively, with no additional wind other than the flow from the gas generator itself. To maintain the temperature in the chamber, a heating cord was wrapped around the exposure chamber (Fig. 1c), and a temperature data logger within a PTFE bag placed in the chamber verified and measured temperature during exposure.

Multiple permeation tubes were used during a given experiment and were grouped based on expected equilibrium time and the temperature that each permeation tube was certified at from the vendor (Fig. 1d). Permeation tubes each contain a single chemical (Fig. 1d), so using multiple permeation tubes allows the testing for multiple chemicals during the same time-series experiment.

**Fig. 2:**
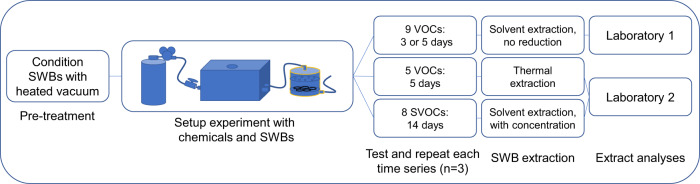
Study design overview. Multiple chemicals and SWBs were used for each time series, and multiple time series was necessary to investigate all compounds and to provide replication. In addition, depending on the set of chemicals, multiple extraction methods and analytical laboratory methods were needed to analyze all target chemicals.

Sets of experiments among compound groups were composed of a single series of 5–6 timepoints over the course of 3–14 days depending on when equilibrium was modeled to occur (Fig. 2). Each time series for each chemical group was repeated days or months apart to provide three true replicates of the time series and avoid pseudoreplication.

### SWB extraction and analytical methodology

Due to the range of chemicals targeted in this research, multiple methods and laboratories were used to quantitate all experimentation (Fig. 2). All SVOC extractions were performed as described previously using two rounds of ethyl acetate, and concentrating the resulting extract to 1 mL/SWB [1]. As appropriate, surrogate standards were added prior to extraction to either monitor extraction efficiencies, or in the case of PAH analyses, correct for losses or enhancement during extraction or from background interference. Some VOC extractions (Table 1) were performed using thermal desorption described in Anderson et al. [34] and used Markes thermal extraction ovens (Markes International, Germany). In contrast to solvent extraction mentioned above, thermal extraction directly volatilizes compounds from the silicone into desorption tubes which are then analyzed. The only deviation from the published thermal methodology was that only a piece of the SWB rather than a whole band was needed for analysis (AVG—0.56 g, relative standard deviation (RSD)—5%).

Other VOC extractions necessitated the use of methanol extracts for analysis (Table 1). These extractions were performed using methanol without a solvent reduction step to prevent losses from evaporation. The method was also optimized to use as little solvent as necessary since the extract would not be concentrated. Each SWB was cut in half (±4%) using a small paper cutter that was solvent-rinsed between samples. Next, solvent-cleaned scissors were used to cut the ½ SWB sample into pieces that would fit into a 4 mL vial. Two rounds of extraction were used for ½ of an SWB (which is approximately equivalent to ≤ 90% of the extractable VOC amount, see Supporting Information and Supplementary Information Fig. 1 inset). Then, 2 mLs of MeOH was added into each 4 mL vial and placed on an orbital shaker for at least 30 min. Afterward, the solvent was decanted into another pre-cleaned and labeled amber vial that could hold both rounds of extraction, and the extraction process was repeated with another 2 mLs of MeOH. After decanting a second time, the original vial with silicone pieces was rinsed with 1 mL of methanol, and the final rinse was added to the rest of the extracted solution for a total of ~5 mLs. Differences in final solvent volume were less than 3% across all samples. These ~5 mL extracts were shipped overnight with icepacks and received by ALS Global (Kelso, WA).

Analytical chemistry and chemical quantitation were performed by outside laboratories using established methods and instrumentation. Specifically, analyses of SVOCs by liquid extraction and thermal desorption was performed by the Food Safety and Environmental Stewardship laboratory at Oregon State University (Laboratory 2 in Fig. 2) [34], and analyses of methanol extracts were performed by ALS Global in Kelso, WA (Laboratory 1 in Fig. 2) using purge-and-trap coupled with GC-MS instrumentation following EPA’s 8260C volatile organics analysis. Both laboratories incorporated analytical QC samples into the analyses according to their respective data quality objectives and SOPs. Additional details can be found in the Supporting Information.

### Modeling

Passive sampling kinetics follow first-order relationships regarding concentrations in the sampler over time [21]. Therefore, a Michaelis–Menton (MM) curve was used in modeling software to describe the first-order relationship in passive uptake, where “*V*_max_” of the curve is analogous to the amount in the sampler at equilibrium (*N*_*s-eq*_). No statistical outliers were justified to remove in uptake curves, so robust nonlinear MM curves were used to predict *N*_*s-eq*_ to reduce, but not eliminate, the influence of individual data points. Modeling of these uptake curves was conducted using R software (Vienna, Austria, https://www.R-project.org/, packages “robust base”) and Graphpad software (Prism 8, San Diego, CA).

Linear models utilized for predicting kinetic parameters log *K*_*sa*_ and log *k*_*e*_ from physicochemical estimates also utilized R and Graphpad software. Where applicable, repeated k-fold CV was used to compare predictive performance among similar models (using ten repeats over ten k-folds) (R, package “caret”).

## Results

### Uptake measurements

**Fig. 3:**
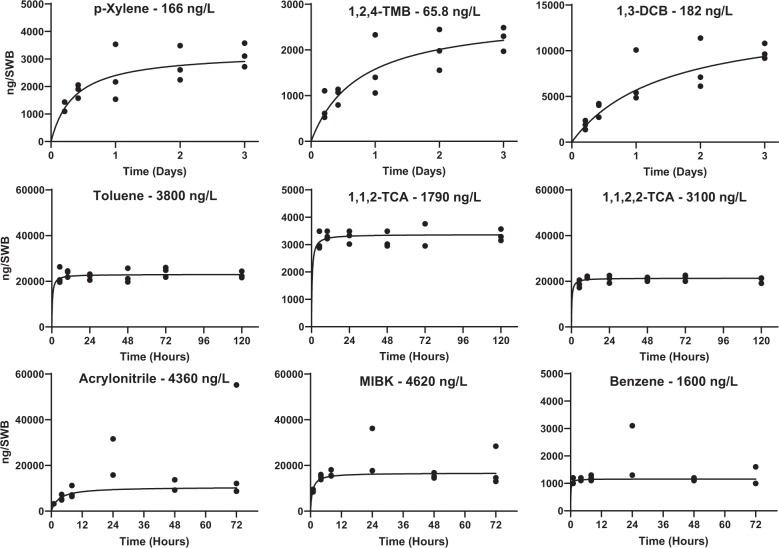
Many compounds established equilibrium over the deployment period. Some were at or close to equilibrium with the first time point. Black lines of best fit are robust regressions of Michaelis–Menton first-order curves.

Uptake curves can be described in three phases: kinetic (linear), intermediate (curvilinear), and equilibrium [21]. In order to calculate a log *K*_*sa*_ parameter, uptake curves must be at or near equilibrium at the end of the exposure period. Of the 22 compounds studied, nine compounds were observed to reach equilibrium within the study period over a range of exposure concentrations (65.8–4620 ng/L, Fig. 3). Most of these compounds reached equilibrium well within the experimental duration, with six compounds near or at equilibrium within the first few timepoints (<12 h, Fig. 3). For XYL, 1,3-DCB, and 1,2,4-TMB, all three stages of the uptake curve were observed over the study period (Fig. 3).

Several other compounds were seen to reach the curvilinear phase (Supplementary Information Fig. 2), including both exposure concentrations of hexachlorobenzene (117 and 0.508 ng/L), as well as undecane and 1,2,4-TCB. For these compounds, a square-root transformation helped to estimate *N*_*s-eq*_ since equilibrium had not been reached yet. Several other compounds (all SVOCs) contained detectable amounts of chemicals from very low concentrations (0.307–4.46 ng/L, or 36.9–505 ppt), but only the kinetic phase was observed (Supplementary Information Fig. 3). It is also unclear if the exposure concentration for these SVOCs was impacted by dry-deposition observed within the delivery system, resulting in lower-than-expected exposure concentrations. What is supported by the SVOC data (specifically all three PAHs, Supplementary Information Fig. 3) is that the SWBs are capable of capturing vapor phase concentrations of SVOCs at low ppt levels (≤36.9–205 ppt). The remaining compounds from Table 1 were not detected, or not detected enough to predict kinetic parameters.

### Measuring and modeling time-based parameters

**Fig. 4:**
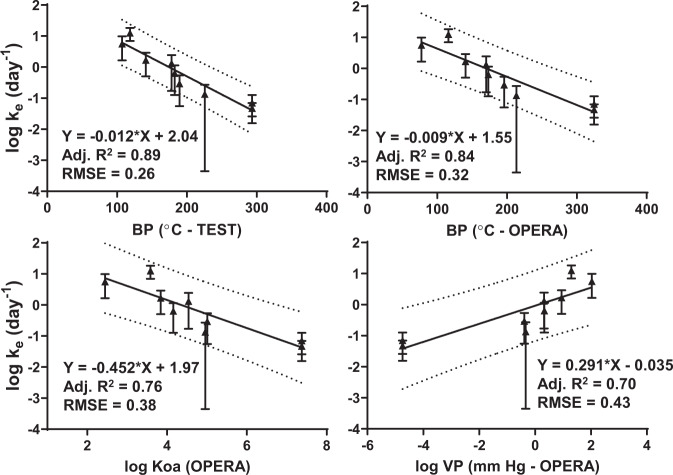
Linear relationships between compound parameters and calculated log *k*_*e*_ values. Lines of best fit are linear least squares regressions. Dotted lines are prediction intervals where 95% of additional points would be expected to occur. Error bars represent 1 SD and include propagation of error.

Uptake data was also used to estimate time-based parameters (i.e., *k*_*e*_ or *R*_*s*_) (see Supporting Information for equations). To estimate log *k*_*e*_ for each compound, the uptake rate (*k*_*u*_) was derived for time points in the kinetic uptake phase of the curve, and then the *k*_*e*_ (dissipation rate) was calculated from the *k*_*u*_ (uptake rate) and *K*_*sa*_ parameters. Once *k*_*e*_ estimates were calculated (see Supplementary Information Table 1 for all parameter estimates), they were log-transformed and compared against various physicochemical characteristics using QSAR-based strategies [35, 36] from EPA’s Comptox database [37] (Fig. 4). Estimates of standard deviation for log *k*_*e*_ included propagation of error (POE) for *k*_*e*_, and then transforming the SDs into log scale (Fig. 4). All four physicochemical characteristics produce good correlations with adjusted *R*^2^ values over 0.7, and root mean squared errors (RMSEs) below 0.5 log units (Fig. 4). The best log *k*_*e*_ model performances utilized boiling point estimates from TEST and OPERA QSAR estimates [35, 36], with >0.8 adjusted *R*^2^, and RMSEs below 0.35 log units (Fig. 4).

### Calculating, modeling, and validating *K*_*sa*_ estimates

After solving for *N*_*s-eq*_ in the MM curves for the 12 compounds reaching or almost reaching equilibrium, *K*_*sa*_ values could be calculated by dividing *N*_*s-eq*_ by the sampler volume (*V*_*s*_) and then by the air concentration (*C*_*a*_) estimated from the Kin-tek (see Supplementary Information for equations and Supplementary Information Table 1). The *K*_*sa*_ values were log-transformed and plotted against physicochemical estimates using the same data sources as mentioned previously (Supplementary Information Fig. 4). All four models (BP-TEST, BP-OPERA, log *K*_*oa*_, and log VP) have adjusted *R*^2^ values ≥ 0.79, and RMSEs under 0.15 log units using weighted (1/*Y*^2^) linear curves. Lines of best fit are weighted since the uncertainty around log *K*_*sa*_ values and equilibrium increases as volatility decreases (i.e., as compounds are less volatile, they become harder to measure in the vapor phase). As seen with log *k*_*e*_ estimates, BP-based models tend to perform better than log *K*_*oa*_ and log VP-based correlations, having a better model fit and lower error (Supplementary Information Fig. 4). However, considering nearly all of this data is from VOC estimates, it is possible that despite the relatively good performance of these models, the observed relationships may be biased for volatile compounds.

**Fig. 5:**
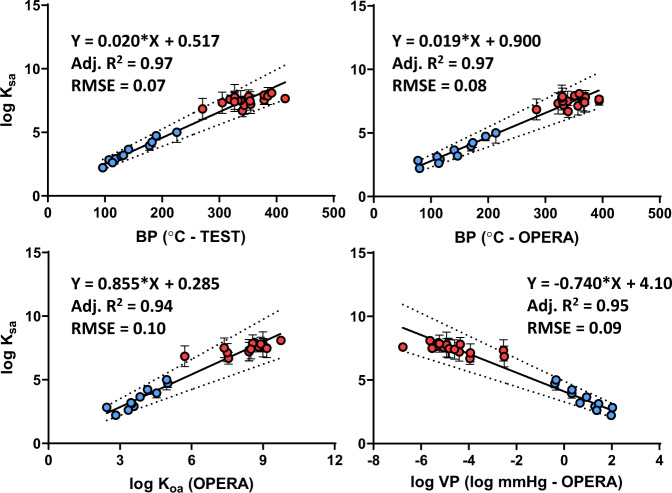
Higher confidence log *K*_*sa*_ data from Tromp et al. [7] (in red) and this study (in blue) graphed among four chemical estimates from EPA’s Comptox database. Lines of best fit are in black and were fitted using unequal weighting (1/*Y*^2^), and dotted lines represent 95% prediction intervals where 95% of future data points are expected to fall.

A paper was published in 2019 that estimated silicone and wristband log *K*_*sa*_ values using only SVOCs under very similar environmental conditions as this study (Tromp et al.~21 °C, ~44% RH, 1.3 m/s wind velocity vs. this study—24.9 °C, 50.4% RH, and <0.15 m/s) [7]. While wind speed is the biggest difference between studies, this factor will more directly impact the rate of uptake parameters (*k*_*e*_ and *R*_*s*_), rather than the ratio of chemicals in the silicone at equilibrium (*K*_*sa*_). Differences in temperature between the studies are just under 5 °C and likely would only affect resulting *K*_*sa*_ values by roughly 0.2–0.3 log units or less based on theoretical estimations of passive air sampling [21, 33]. Because these changes would be expected to be much less than the variance already reported in the majority of Tromp et al. data, no temperature adjustment was performed. Overall, the similarity in conditions between studies makes it possible to combine *K*_*sa*_ data from this previous research with the current study. Using all log *K*_*sa*_ values from Tromp et al. coupled with this study’s estimates of log *K*_*sa*_ (a total of 87–89 compounds including VOCs and SVOCs, and a log *K*_*oa*_ range of 2.44–11.8), models were created again using BPs, log *K*_*oa*_, and log VP (Supplementary Information Fig. 5). The differences between model performances are now less pronounced, although BP TEST still has the highest adjusted *R*^2^ and lowest RMSE (0.89 and 0.11 respectively). In looking at the data in Supplementary Information Fig. 5, it is clear that many log *K*_*sa*_ estimates have quite large SDs (over 1 log unit). Since 1 SD in log scale already represents an order-of-magnitude difference in an environmental concentration, a conservative limit of 1 SD was applied to both datasets to utilize *K*_*sa*_ estimates with relatively low uncertainty (< 1 log unit SD). Removing this uncertain data from both datasets can help further refine the predictive performance of each physicochemical estimate and still maintain chemical diversity (*n* = 29 chemicals of different classes, VOCs and SVOCs, and a log *K*_*oa*_ range of 2.44–9.74). In Fig. 5, selected log *K*_*sa*_ data that had less uncertainty (< 1 log unit SD, referred to as higher confidence data, or HCD hereafter) were graphed again for all four models for a final set of models.

After using data with less uncertainty, HCD model performance increased from the full datasets (see Fig. 5 vs. Supplementary Information Fig. 5). All four HCD models have *R*^2^ values ≥ 0.94, and RMSEs ≤ 0.11. The differences in adjusted *R*^2^ and RMSE are less pronounced between models, although BP-based models again have marginally improved outcomes over log *K*_*oa*_ and log VP estimates. Considering the relative weight and impact log *K*_*sa*_ has on calculating air equivalency, the results in Fig. 5 increase the likelihood of accurate predictions for air equivalency across the range of chemicals in each model. Also, it is interesting to note that each equation has altered only slightly from the original set of modeling based on this study’s data (see equations in Fig. 5 vs. Supplementary Information Fig. 5). In fact, if all of the lines of best fit are compared as data is added, refined, or weighted, the slopes and intercepts do not change dramatically, with BP-OPERA changing the most once Tromp et al. [7] data are added (see Supplementary Information Fig. 6). The result that the equations are not changing dramatically between most refinements represents a level convergence among models, which increases the confidence in this dataset. However, it is important to have a sense of predictive power before comparing log *K*_*sa*_ estimates with published data. Cross-validation (CV) is a way to gauge the performance of these models and identify the most accurate and least variable model moving forward.

A repeated k-fold CV (ten repeated CVs on ten k-folds) was performed for each of the models using the full and the HCD models to evaluate model performance and any equation changes (Supplementary Information Table 2). The results of the models do not reveal large differences in *R*^2^, RMSE, or mean absolute error outcomes from each CV series, but generally log VP-based models tend to perform the worst across metrics, while BP-TEST-based models tend to perform the best overall (Supplementary Information Table 2). BP-OPERA and log *K*_*oa*_ performances tend to be similar, with log *K*_*oa*_ slightly better at CV outcomes when considering the full datasets but BP-OPERA becoming closer to log *K*_*oa*_ when considering the HCD models (Supplementary Information Table 2). When examining CV changes in equations from the original models, the largest differences are in the intercepts (both sets of equations are given in Supplementary Information Table 2). Often the differences in intercept are less than 0.2 log units in full models, and less than 0.1 log units in higher confidence datasets (HCD models). Despite these small differences, because changes are in log scale it is recommended to use the final HCD equations from the CV results from Supplementary Information Table 2 shown here:

Eq. (3) (inputs of physicochemical parameters are from the EPA Comptox Database [37]): BP-TEST (CV): log *K*_*sa*_ = 0.020^*^(BP–°C) + 0.527; *Adj. R*^*2*^ *=* 0.97, CV RMSE *=* 0.445, *N* *=* 29.

Eq. (4) (inputs of physicochemical parameters are from the EPA Comptox Database [37]): BP-OPERA (CV): log *K*_*sa*_ = 0.019^*^(BP–°C) + 0.829; *Adj. R*^*2*^ *=* 0.97, CV RMSE = 0.436, *N* *=* 29.

Eq. (5) (inputs of physicochemical parameters are from the EPA Comptox Database [37]): Log *K*_*oa*_-OPERA (CV): log *K*_*sa*_ = 0.867^*^(log *K*_*oa*_) + 0.190; *Adj. R*^*2*^ = 0.95, CV RMSE = 0.453, *N* *=* 29.

### Air equivalency calculations

Using the best performing sets of models for *k*_*e*_ and log *K*_*sa*_, a final comparison of air equivalency was performed using the two HCD BP log *K*_*sa*_ models (Eqs. (3) and (4)) and their corresponding log *k*_*e*_ models in Fig. 4. Using arbitrary inputs of sampler volume (*V*_*s*_), the amount of chemical found for a deployed SWB (*N*), and time-worn (*t*), the resulting air equivalency (ng/L) was estimated using appropriate chemical BPs (TEST or OPERA) for all compounds in Table 1. Agreement between the two models was often within twofold (13 out of 21 comparisons) and had differences of 2.5–5-fold once values were below 1 ng/L (representing less volatile SVOC compounds, see Supplementary Information Table 3). Air equivalency estimates incorporate differences from each set of models in both log *K*_*sa*_ and log *k*_*e*_ estimates, but a Spearman’s r correlation was still >0.98 over 5 orders of magnitude, with most estimates between sets of models close to a 1:1 correlation line (Supplementary Information Fig. 7). Given the consistency of the ng/L estimates and performance of these models, it is recommended to use BP-TEST-based models (Eq. (3) and associated equation for log *k*_*e*_ in Fig. 4). If a QSAR BP-TEST value is unavailable for that chemical, then it is recommended to use BP-OPERA-based models secondarily.

## Discussion

### Parameter modeling and comparisons

This study improves upon previous estimates of partitioning parameters that help calculate air equivalent data from silicone personal samplers and uses a novel physicochemical characteristic that has not previously been shown to help predict air concentrations. Existing methods and equations for estimating sampling rate parameters and/or log *K*_*sa*_ for silicone [3, 7, 34, 38] do not use chemical BPs for modeling these parameters. However, BPs have been used to model a rate-based uptake parameter with permeation samplers with excellent correlations among several VOC compounds (*R*^2^ = 0.89) [30]. While not initially intuitive, chemical BPs are a function of intermolecular forces, and intermolecular forces are also responsible for passive sampling interactions between chemicals and the polymer backbone of sampling material. Furthermore, correlations between thermodynamic properties (i.e., enthalpy of sublimation and vaporization) and melting or BPs have been observed previously [39]. Finally, since BPs are readily available for most compounds (experimental and/or predicted with QSAR) and rarely differ as dramatically among in silico and experimental results as some other parameters often presented in log-scale, this physicochemical characteristic is simple and effective for use in passive sampling kinetic modeling.

Prior to this study one of the most comprehensive investigations of chemical uptake into silicone utilized linear free energy relationships (LFER) to create *K*_*sa*_ models using over 140 compounds [38]. However, there are not always LFER estimates for every chemical, which decreases the utility of this method and associated models. Recently, Tromp et al. [7] provided a relatively simpler process and equation to estimate *K*_*sa*_ with just one input (log *K*_*oa*_) for PDMS but used log *K*_*oa*_ values from other publications that are again not available for many compounds [7]. This study addresses these issues by offering sets of straightforward empirically-based linear models based on widely available BPs to estimate uptake parameters necessary to estimate air equivalent concentrations.

In addition, the simplified HCD models estimating log *K*_*sa*_ values generated in this study compare well to other published methods. Log *K*_*sa*_ estimates including all the compounds in Table 1 and the models mentioned above along with another model that incorporated SWBs in real-world environments are compared in Supplementary Information Table 4 (nine estimates for each chemical in total). Generally, the difference between all estimates is less than 1 log unit for VOCs (excluding DCM which has a 1.18 log difference between lowest and highest estimate) but differs more for SVOCs (1.87–5.24 log differences across nine estimates). Refining comparisons further to only models with *R*^2^ ≥ 0.95 five models total—3 HCD models from this study, Tromp et al. [7] and Sprunger et al. [28] estimates of log *K*_*sa*_ for compounds in Table 1 are within a log unit for 15 out of 22 chemicals (Supplementary Information Fig. 8). Again, the largest differences among models are at either end of volatility extremes, with the largest differences for SVOCs (1.03–2.97 log unit differences) which is expected, since certainty and relevancy around estimates of air concentration exposures decrease as volatility decreases.

Exploring log *K*_*sa*_ comparisons with Sprunger et al. [28] further, a much larger internal dataset of chemicals detected in SWBs were utilized (185–190 possible comparisons including VOCs and SVOCs), and there are excellent correlations between models (Supplementary Information Fig. 9). Among these correlations, BP-TEST has a Spearman’s *r* = 0.97, and BP-OPERA and log *K*_*oa*_-OPERA models have *r* = 0.94 coefficients (Supplementary Information Fig. 9). Deviations from a 1:1 relationship from LFER log *K*_*sa*_ estimates increased around log *K*_*sa*_ values of 8 and higher, with increasing deviations as estimates increased in value (Supplementary Information Fig. 9). Again, this is expected since atmospheric-based measurements of chemicals will inherently be harder to detect and model with accuracy as volatility decreases. Ultimately, the overall agreement between models estimating log *K*_*sa*_ values for PDMS or SWBs is promising since the accuracy of this value is the most critical to the accuracy of air equivalent calculations (see Supplementary Information—passive sampling theory and equations).

The purpose of estimating log *k*_*e*_ and log *K*_*sa*_ values is so that they can be used to estimate air equivalency concentrations from real-world deployments. Considering that much of an individual’s time is spent indoors (≥80% in some cases [40]), the assumptions of environmental conditions in this study are consistent with many real-world scenarios and enables studies using the equations described herein to normalize extract data to make comparisons about exposure. In applications clearly outside of these environmental conditions, it is possible to adjust log *K*_*sa*_ values for temperature, although specific relationships for temperature adjustments are currently only available for PUF personal samplers (roughly 0.03–0.06 log units per 1 °C) [33]. For rate-based parameters like *k*_*e*_ and *R*_*s*_, using performance reference compounds (PRCs) is ideal, since rate-related uptake is determined in situ, rather than estimated [21, 23]. However, this approach can be challenging when determining which compounds and at what levels to infuse into the wristband prior to deployment considering the safety of individuals wearing the passive sampler. Ideally, any infused levels of PRCs would be below ambient levels of target compounds previously reported in SWBs. Since this study attempts to simply estimate the rate-based uptake, there is no need for this extra calculation, but necessitates the use of “equivalency” since all resulting air concentrations have environmental assumptions.

### Additional air concentration limitations and future directions

Aside from the assumptions regarding environmental conditions and equivalency outlined above it is important to consider the relevancy of atmospheric values regarding the potential routes of exposure. SWBs are capable of sequestering compounds not only from atmospheric uptake but also from other sources including direct or dermal contact [5, 20]. For some compounds, it is much more likely that concentrations seen in SWB extracts are due to direct contact (e.g., particle scavenging, material surfaces like carpet, or through skin contact) rather than atmospheric [5]. Thus, the larger the log *K*_*sa*_, not only is the estimate more likely to have more uncertainty but the relevancy of air concentration data is often reduced. Another consideration when calculating air equivalent concentrations is degradation. Interpretation of parent or derivative compounds should be made in context not only with the environment the sampler was worn but also in regard to the sampler time and half-life of the target compound to ensure the relevancy of the environmental estimate.

To address questions of relevancy of air equivalency data, future investigations of SWBs (or other silicone samplers) should explore routes of exposure further considering a wide range of chemical diversity, especially around a log *K*_*sa*_ of 8 since error and uncertainty increase dramatically above this value (Supplementary Information Fig. 9). It is likely that estimates of dominant routes of exposure could be modeled against physicochemical characteristics similar to what was modeled here, and studies may even include surrogate skin uptake experimentation, or combinations of samplers that isolate routes of exposure such as previous studies [5, 9, 10]. This would help add additional context and comparability to air equivalent data from personal silicone samplers. Similarly, an expansion of this research could include changes in environmental factors such as temperature, humidity, and windspeed so that mathematical adjustments could be made to alter *K*_*sa*_ and rate-based parameters using empirical measurements and modeling as appropriate.

Another potential avenue of research can further explore the links between silicone extract concentrations (regardless of route of exposure) and internal biomarkers [17–19], establishing relationships between exposure levels and internal levels. This could be especially helpful if using SWBs as a screening tool before more expensive or complex analyses. Finally, utilizing the data outcomes from this study, comparisons of atmospheric equivalencies can be made with other passive or active sampling devices to evaluate the performance of both the approach as well as the calculations from this study when utilizing SWBs for personal chemical exposure.

## Supplementary Information

Supporting Information
