# Supplementary material for: Determining chemical air equivalency using silicone personal monitors
Source: J Expo Sci Environ Epidemiol. 2021 May 5;32(2):268–79. doi: 10.1038/s41370-021-00332-6 (PMC8920887; doi:10.1038/s41370-021-00332-6)
Supplement: Supplementary file 1 — Supporting Information [file 41370_2021_332_MOESM1_ESM.docx]

Supporting Information:

# Determining chemical air equivalency using silicone personal monitors

S. O’Connell, K. Anderson, and M. Epstein

Contents

[Passive sampling theory 2](#_Toc57626203)

[Methanol extraction for VOCs 3](#_Toc57626204)

[Analytical chemistry details 4](#_Toc57626205)

[FSES Laboratory 4](#_Toc57626206)

[ALS Global 5](#_Toc57626207)

[Raw and transformed uptake data 6](#_Toc57626208)

[Uptake estimates from chamber experiments 7](#_Toc57626209)

[Combining data from this study and Tromp et. al., 2019 9](#_Toc57626210)

[Lines of best fit for Log K_sa_ relationships 10](#_Toc57626211)

[Model performance and comparisons 11](#_Toc57626212)

[Cross-validation 11](#_Toc57626213)

[Example of air equivalency calculation 11](#_Toc57626214)

[Comparison of log K_sa_ values with other publications 13](#_Toc57626215)

[Correlation of higher predictive HCD models with Sprunger et al., 2007 LFER estimates 15](#_Toc57626216)

[References 17](#_Toc57626217)

## Passive sampling theory

For passive samplers like SWBs, a dose-response relationship between the amount absorbed into the wristband and an increase in that amount over time given constant exposure is expected to follow a first-order model in 3 stages: kinetic (linear), intermediate (nonlinear or curvilinear), and equilibrium ^1,2^. This relationship can be modeled from a Michaelis-Menton first-order relationship to provide a line of best fit for compound concentrations in the sampler over time.

The expected maximum concentration of chemicals in silicone at equilibrium (C_s-max_) can be calculated once equilibrium is reached or estimated if the uptake curve is near equilibrium. This C_s-max_ represents the amount (N_s-eq_) found in the wristband divided by the size of the sampler, in this case, the volume of the wristband (V_s_). Since the air concentration (C_a_) is known from the vapor generator system and certified permeation tubes, the C_a_ can be used to estimate the partition coefficient (K_sa_), which is the ratio of the maximum equilibrium concentration in the silicone (C_s-max_ - ng/L) over the air concentration (C_a_ - ng/L) expressed in Eq. 1. The partition coefficient between the sampler and the environment is the single-most influential parameter to identify (mathematically) in order to improve the accuracy of estimating air concentration from passive samplers. Especially since this parameter is often expressed on a log scale due to the large differences in K_sa_ between compounds.

$$Eq 1. K_{sa}=\frac{C_{s-max}}{C_{a}}$$

For SWBs that are regular-sized, the wristband volume is approximately 0.00445 L of silicone (V_s_), and concentrations of target compounds are calculated in ng/L (C_s_). If measurements of uptake included the linear kinetic phase of uptake, sampling rates (R_s_) and dissipation rates (k_e_) values can also be estimated using the following equations:

$$Eq 2. k_{e}= \frac{C_{s}}{C_{a}\times t\times K_{sa}}$$

$$Eq 3. R_{s} = \frac{N}{C_{a}\times t}$$

where *t* is time in days and *N* is the amount in the sampler (i.e. the amount (ng) in the entire WB, not normalized by volume). In passive sampling theory, the relationship between dissipation and uptake rates are expected to be inversely related ^1,3,4^, so the inverse relationship of the uptake of target compounds in the chamber experiments can be used to estimate this dissipation rate, *k_e_* from Eq 2. The parameter k_e_ is normalized by K_sa_ in Eq 2., which helps model this parameter to physicochemical characteristics and this modeling is depicted in the manuscript.

It is important to note that $K_{sa}$ , R_s_, and *k_e_* can change depending on environmental conditions, and are not set constants for each chemical regardless of deployment. However, since many personal exposures can happen indoors, the uptake experiments were performed at approximately 25°C and 50% humidity to simulate real-world conditions that are standardized. Since R_s_ and k_e_ are related (k_e_ = R_s_/(V_s_*K_sa_)), either parameter can be used to estimate C_a_ if all other parameters are known so that silicone wristband concentrations can be converted into atmospheric equivalent concentrations (assuming 25°C and 50% humidity, see Eqs. 4 and 5 for C_a_ equations based on k_e_ and R_s_ respectively).

$Eq\mathbf{.} 4\mathbf{:} C_{a}=\frac{N_{compound}}{V_{s}{\times K}_{sa}\times(1-e^{(-k_{e}\times t)})}$

or

*Eq*. 5: $C_{a}=\frac{N_{compound}}{V_{s}{\times K}_{sa}\times(1-e^{(-\frac{R_{s}\times t}{V_{s}\times K_{sa}})})}$

More information about the equations and theory presented here can be found in Chapter 3 of Huckins et al., 2006 (1) or Vrana et al., 2005 ^5^.

## Methanol extraction for VOCs

All VOC extractions were performed through an adapted solvent extraction without a solvent reduction step to prevent loss of compounds during processing. Also, the method was optimized to use as little solvent as necessary to reduce the dilution of the sample extract. To demonstrate this method before use on samples described in the chamber experiments, a simple extraction experiment was conducted to evaluate the performance of this method. First, VOCs were infused into four replicate SWBs. Briefly, a 55-compound VOC mix (Accustandard) was pipetted into an amber glass vial containing a full regular-sized wristband. Then, after letting SWBs equilibrate with compounds for at least 2 hours at 30°C, the wristbands were stored in airtight PTFE storage bags in the freezer at -20°C. Then these infused SWBs could be used to evaluate the extract method, with four replicates for each round of extraction. The VOC-infused SWBs were extracted with three rounds of MeOH, using only five mLs for each round by using small vials (approx. 9 mLs) and cutting the wristbands into four pieces and inserting them into the vial with solvent-rinsed tweezers. These extracts were packaged with ice packs and shipped to ALS Global for VOC analyses.

In SI-Figure 1, the percent of the total amount extracted in each extract are shown in stacked bar graphs, with the compounds in ascending order of boiling point (i.e. volatility) from left to right on the x-axis. All compounds analyzed by the method were able to be taken up and measured in each round of extraction (SI-Figure 1). Clearly, the first round of extraction is responsible for most of the removal of all VOC compounds from the silicone (see left hand graph, black bars), which is expected and seen in other silicone extraction data ^6^.

The other conclusion from this evaluation is seen in the right-hand graph of SI-Figure 1, which shows the overall percent extracted for all VOCs from the 1^st^, 2^nd^, and 3^rd^ rounds of extraction normalized for the total amount. The initial round of extraction accounts for an average over all the VOCs of 71% (+/- 7% SD) of the total amount extracted. The 2^nd^ round accounts for an average of 22% (+/- 4% SD) across all the VOCs analyzed, and the 3^rd^ round, 8% (+/- 3% SD). Given that these samples will not be blown down and concentrated, the optimal strategy if extracting an entire wristband using this methodology is to simply use one round of extraction since the potential benefit of a second round (i.e. 22%) is less than the detriment of another round of extraction which dilutes the sample (50% lower). Given the small amount of extracted amounts in the third round of extraction along with the risks of contamination and diluting the sample further, it is not recommended to utilize three rounds of extraction.


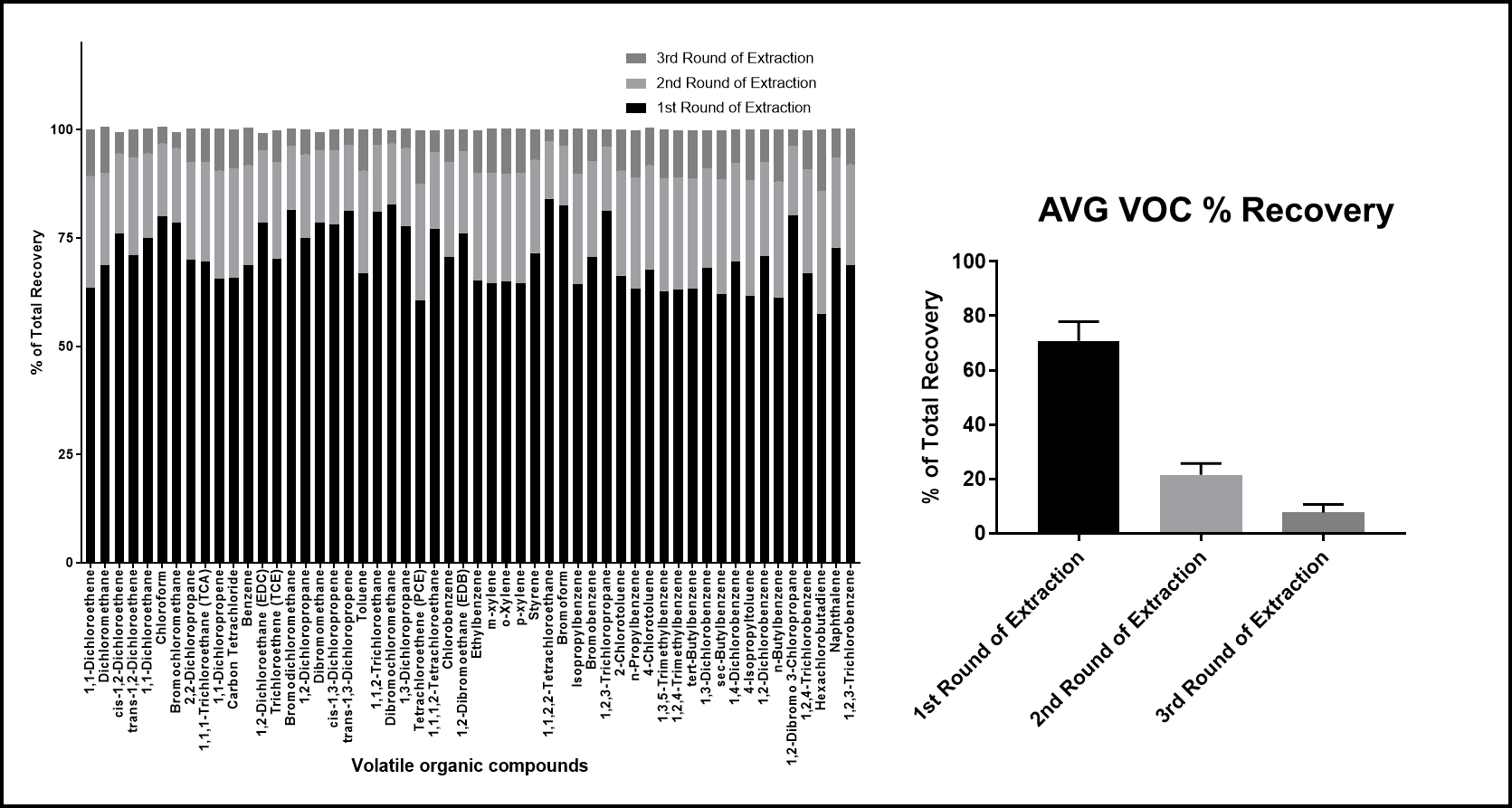


SI-Figure 1. In the left-hand graph, averaged individual VOC recovery amounts normalized to the total amount extracted from 3 rounds per SWB is depicted (n=4). The right-hand graph depicts the overall average VOC recovery percentage per round of extraction. Note that m- and p-xylenes were depicted here after halving the values since they co-elute in this analytical method. They should be interpreted as approximate values.

## Analytical chemistry details

### FSES Laboratory

For SVOCs, several analytical methods were used based on established analytical methods for different compound classes. Briefly, a gas chromatograph (GC) with an Agilent DB-5 column (30 m length, 0.25 mm inner diameter, 0.25 µm film thickness) was used to chromatographically separate carbonyls and flame retardants, while an Agilent DB-XLB (30m, 0.25mm, 0.25 µm) and a DB-17MS (30m, 0.25mm, 0.25 µm) was used to separate organophosphates, nitramines, pyrethroids, nitroaromatics, and organochlorines with dual column confirmation as previously described ^7^. An Agilent Select PAH column (30m, 0.25mm, 0.15 µm) was used for PAH separation. Analyses of SVOCs used mass spectrometry (MS) detection (model 5975B, Agilent) for flame retardants, dual electron capture detection (ECD, model 6890N, Agilent) for pesticides, or for PAHs, a tandem mass spectrometry system was used as described previously for all detection methods ^8^.

For some VOCs (see Table 1), thermal desorption was used directly on pieces of SWBs. A Markes tube conditioner (TC-20), a thermal desorption unit (Micro chamber/thermal extractor M-CTE-25), and a thermal desorption auto-sampler (Series 2 Ultra) and thermal tube-desorber (Unity 2) directly plumbed to an Agilent GCMS (5975B) were used to condition tubes, extract silicone onto C3-AAXX-5304 thermal desorption (TD) tubes and analyze the extracted desorbed air from SWBs respectively. Oven profiles, injection parameters, instrument internal standards, and surrogate standards for each method have been described previously ^8^.

Each method had unique data quality objectives (DQOs). Calibration standards were run prior to any samples to the accuracy of analytes using the setup and system. During the run, instrument blanks were used every 10-30 samples, between batches, at the start of batches, and after maintenance to ensure system contamination was not present or adversely affecting quantitation. A calibration standard was also analyzed every 20 samples and/or at the end of every batch of samples. If a calibration standard failed to meet DQOs, system maintenance was performed until standards could be ran and analyzed that could meet DQOs.

### ALS Global

Methanol extract analyses ran by ALS Global were conducted according to EPA 8260C (VOCs by GC-MS) methodology using a purge-and trap setup to measure analytes from 0.5 mLs of sample solution. Briefly, 0.5 mLs were diluted into 50 mL of ultra-pure water and helium was used as the carrier gas through the solution and collected using a purge and trap system with a gas flow rate of 40 mL/min, purged for 11 min., desorbed for 2 minutes at 240°C, and baked at 260°C for 20 minutes (Tekmar 3000 or equivalent), and then separated and analyzed using GC-MS instrumentation (Agilent MSD, Chemstation software, RTX-624 column). QC samples included lab control samples, duplicate lab control samples and method blanks from ALS Global, and also included positive and negative controls as part of the study design and set of samples sent out to the laboratory. All QC samples passed data quality objectives of recovery, background, and duplicate performance, except for MEK background. After background correction it was determined that MEK could not be reliably reported in this dataset.

## Raw and transformed uptake data

Hexachlorobenzene, 1,2,4-trichlorobenzene, and undecane were detected in all time points, but did not approach equilibrium during the exposure period. However, these compounds did approach or reach the nonlinear or intermediate state of uptake (SI-Figure 2). A square-root transformation was performed on the raw data to attempt to better estimate a partition coefficient for each compound. A square-root transformation is a less-drastic form of transforming the data than a log-transformation which would too strongly affect the prediction of the first-order uptake model.

The first uptake chamber experiment used hexachlorobenzene (HCB) at 117 ng/L, or 10 parts-per-billion (ppb). However, this concentration ended up being too high for the calibration of the analytical method. As seen in SI-Figure 2A, a 10-ppb air concentration of HCB resulted in 100,000s of ng in each wristband at a given timepoint. Subsequent SVOC experiments utilized a dilution module for the Kin-tek that provided a diluted flow resulting in parts-per-trillion exposure levels (see lower set of graphs on left hand side of SI-Figure 2A). Even though experiments were performed for HCB at drastically different concentrations (nearly 1000-fold), estimates of partition coefficients were within 0.7 log units (6.11 vs 5.48).

For 1,2,4-TCB and Undecane, curves of square-root transformed data helped estimate log K_sa_ estimates, and timepoints that were in the kinetic uptake phase were used to help estimate k_e_ and R_s_ values of rate-based parameters of uptake based on Eqs. 2 and 3 respectively.

SI-Figure 2. Uptake data for compounds nearly reaching equilibrium. Estimates of K_sa_ were determined through square-root transformations. All curves used robust linear regression to create the line of best fit.


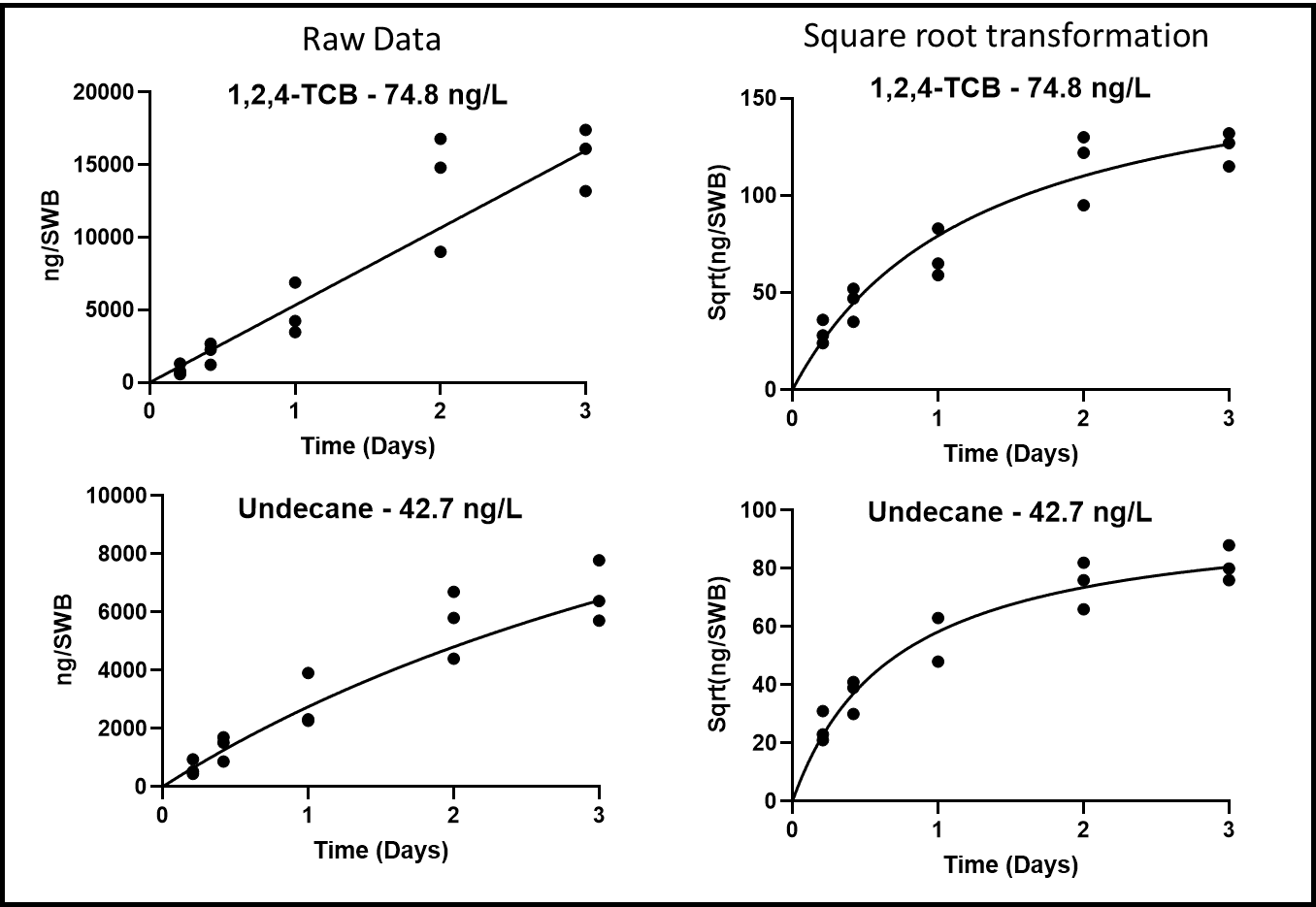

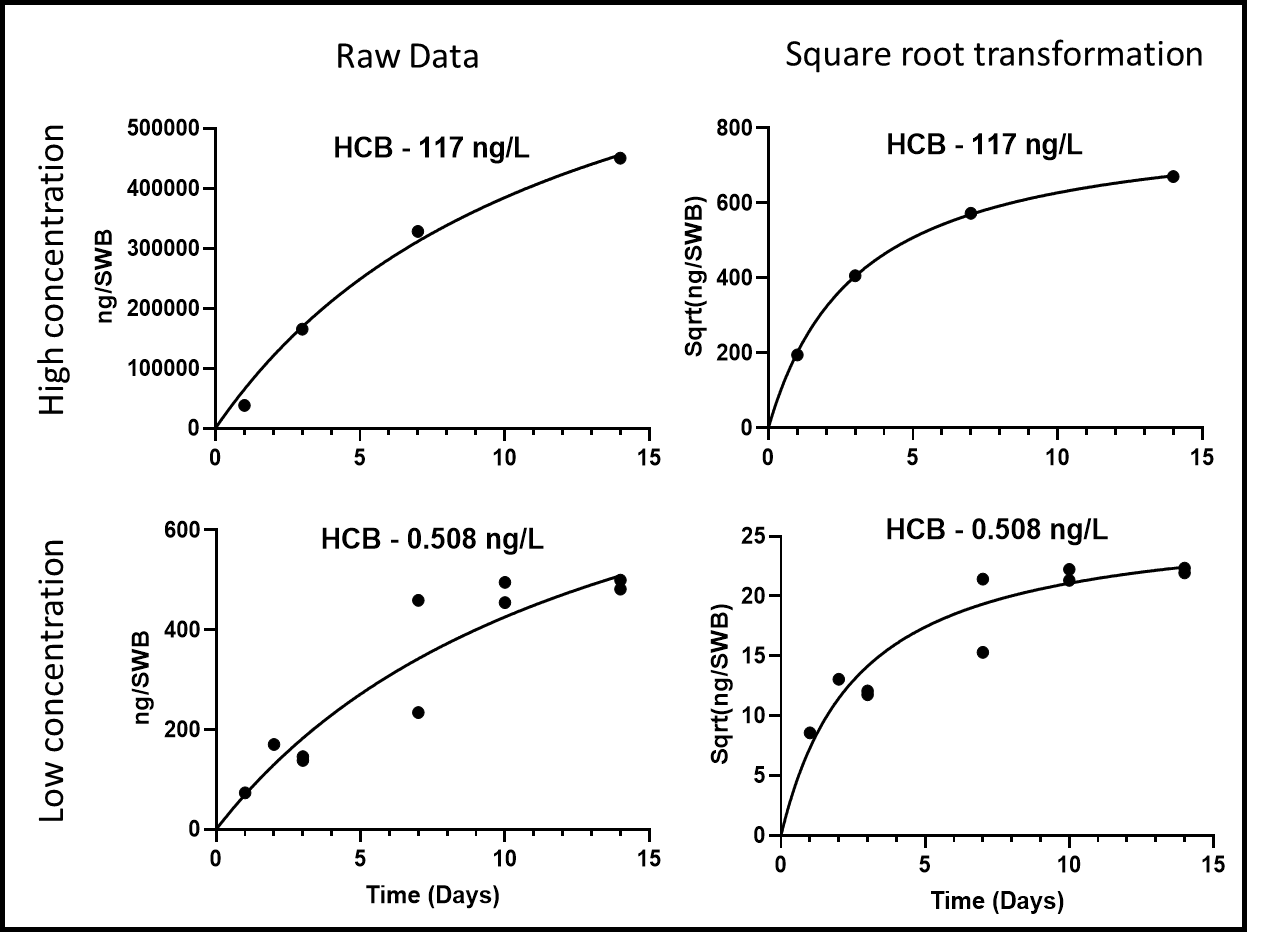


**2A**

**2B**

SI-Figure 3. Uptake data for compounds not close to equilibrium, or too variable to estimate uptake (2,4-DNT).

All three PAHs were detected at all time points in the linear uptake phase (SI-Figure 3). However, PAHs had levels of concentrations far lower than expected uptake amounts after earlier experimentation. A likely explanation is that these compounds were not delivered by the Kin-tek equipment to the wristbands as designed. This hypothesis is supported by visual evidence of compound deposits in the main PTFE tubing prior to the first mass-flow controller on the Kin-tek, which was not entirely covered by heated cord like the rest of the tubing. Afterwards, the entire gas-vapor system was sent back for maintenance, and to insulate that section of tubing. Regardless, PAHs are possibly not volatile enough to be measured accurately using this setup and were excluded from any further modeling.

The chemical 2,4-DNT was detected in all experiments with SWBs but was too variable to report and predict a partition coefficient (SI-Figure 3). It could stem from reasons outlined above, from variability in the analytical method, or ultimately variability in the uptake of this compound into the silicone, but further testing would be warranted to draw a stronger conclusion. Permethrin, chlorpyrifos, and TCEP were not detected in the samples from any of three experimental replicates. All three of these compounds have a predicted higher K_sa_ than phenanthrene, so they are more likely to have deposits in the same section of tubing as the PAHs regardless of insulation.

Ultimately, what was discovered from the SVOC analyses were partition estimates from HCB and the discovery that the wristbands can absorb SVOCs from the atmosphere at extremely low concentrations (parts-per-trillion). Additionally, implications of the difficulties of vaporization and deposition of some of the higher MW and low-volatile compounds could indicate that deployments that result in the detection of these compounds in SWBs might not primarily stem from atmospheric exposures, but from other routes of exposure, as highlighted in other publications ^9–11^ and discussed further in the limitations and future directions section of this manuscript.

## Uptake estimates from chamber experiments

Once parameters such as boiling point (BP), octanol-air partitioning coefficients (K_oa_), and vapor pressure (mmHg @ 25°C) were acquired using the EPA Comptox database (<https://comptox.epa.gov/dashboard>), the logarithmic silicone-air partition coefficients (log K_sa_) were modeled as weighted (1/Y^2^) linear correlations and graphed (SI-Figure 4). In each graph, adjusted R^2^ and Root Mean Square Error (RMSE) are given for each model to help compare the performance of each model with one another. RMSE values are in the same units as the y variable, so even small differences between models can mean a much better prediction of log K_sa_. From SI-Figure 4, several physicochemical parameters appear to be very predictive (≥ 0.79 – Adj. R^2^, and less than 0.13, RMSE) of the partitioning coefficient, log K_sa_. In particular, BP estimates have strong correlations (Adj. R^2^ ≥ 0.87) and lower error on a log scale (RMSE ≤ 0.1).


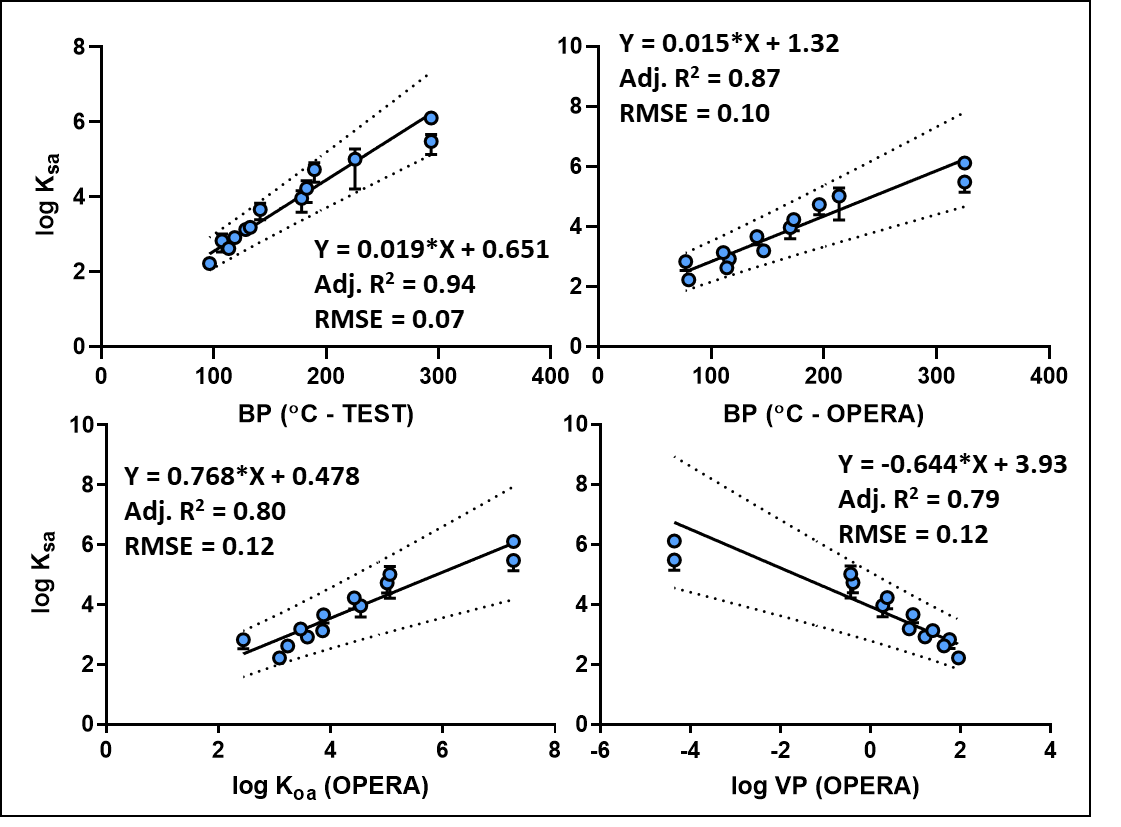


SI-Figure 4. Linear regressions between log K_sa_ values determined in this study with physicochemical parameters. Error bars represent 1 SD. OPERA or TEST designate the source of the QSAR generated parameter from EPA’s Comptox Database. Regressions are weighted (1/Y^2^), and dotted lines represent 95% prediction intervals.

Once log K_sa_’s were estimated from the raw and transformed data using MM robust curves and equation 1, other parameters could be estimated using equations 2 and 3. Additionally, estimates of equilibrium time can be simply calculated using the half-time to equilibrium equation times four ^1^: Equilibrium estimate = (LN(2)/k_e_)*4. All of these parameter estimates can be found in SI-Table 1 below.

SI-Table 1. Uptake parameters generated using this study's Kin-tek and custom exposure chamber experiments

^Denotes that a square root transformation was used to estimate Log K_sa_

*Values are approximate, the time point used for this estimate was already at equilibrium. These values were not used in model building.

One overall finding from SI-Table 1 is that many VOCs reach equilibrium quickly (less than 1 day), and that is especially true for compounds with MW less than 100 g/mol or have BPs below 100°C. Reaching equilibrium does not mean that the SWBs are ineffective, and in fact, some passive samplers are designed to come to equilibrium quickly (ex. silicone fibers used for solid-phase microextraction, or SPMEs). However, it does imply that VOCs measured from SWBs may represent a much shorter time period of exposure than the overall deployment period of a project or application. For example, a SWB that measures toluene from a 7 or 30-day exposure may only represent the average exposure over the last day of that deployment and not a time-weighted average over the whole period like measurements from hexachlorobenzene or phenanthrene would represent (see equilibrium time estimates in SI-Table 1). Of course, this is assuming the exposure is solely from the atmosphere, and not from particle scavenging, dermal exposure, or through direct contact. Regardless of the stage of uptake, air equivalency calculations of air concentrations (C_a_) listed in equations 4 and 5 does not suffer from bias in the calculation regarding uptake stage, and both equations are appropriate to use whether a compound is at equilibrium or kinetic uptake phases ^1^.

## Combining data from this study and Tromp et. al., 2019

In 2019, a study was published attempting to measure the dissipation and uptake of over 200 SVOC compounds into silicone wristbands and other passive sampling media over 6 months in atmospheric conditions similar to those used in this study ^4^. Conditions of both laboratory experiments from Tromp et. al and this study were similar in averaged temperature and humidity (21°C and 44% RH in Tromp et al., and 25°C and 50% RH in this SBIR). There were differences in wind speed (1.3 m/s vs <0.15 m/s), but this difference is more likely to lead to significant differences in uptake rates rather than amounts at equilibrium (i.e. log K_sa_). Using measured log K_sa_’s and SDs published in Tromp et al., 2019 combined with those from this study, the following relationships can be shown using the same physiochemical parameters and data sources used previously (SI-Figure 5).


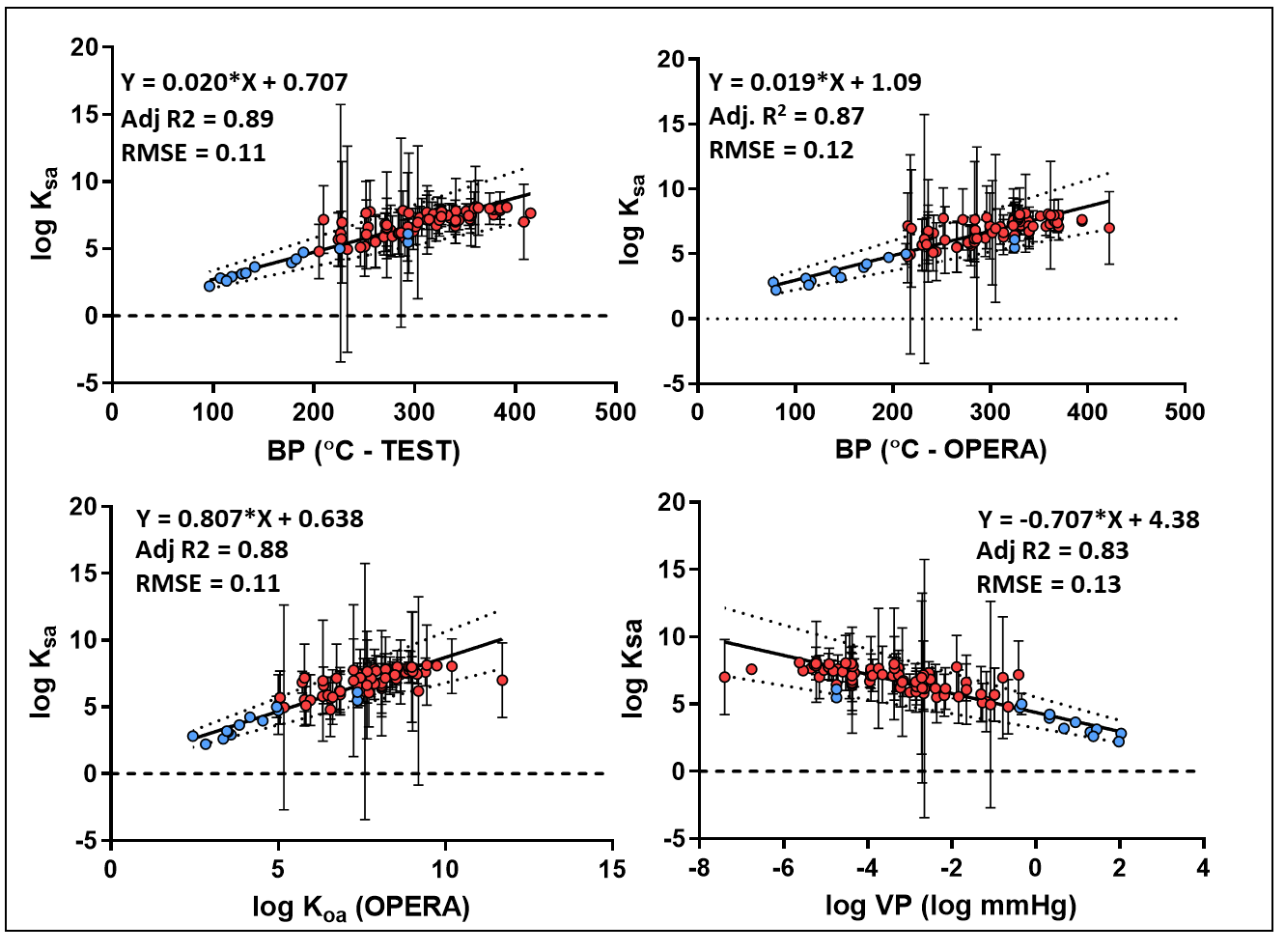


SI-Figure 5. Linear regressions of log K_sa_ and chemical parameters using this study and Tromp et al., 2019 data. Error bars represent 1 SD. OPERA or TEST designate the source of the QSAR generated parameter from EPA’s Comptox Database. Regressions are weighted (1/Y^2^), and dotted lines represent 95% prediction intervals. Blue dots represent data from this study, while red dots represent Tromp et al., 2019 data.

The data shown in SI-Figure 5 are much different than those published in Tromp et al., 2019. However, this is due to the authors of that manuscript selectively plotting a number of PAHs, PCBs, and OCPs instead of the full dataset based on the source of their log K_oa_ values. Regardless, it is clear from SI-Figure 5 that there are relationships between boiling points (whether from TEST or OPERA sources), log K_oa_, and log VP with log K_sa_ estimates (all R^2^ > 0.80). Also of note, the final equation given in Tromp et al., 2019 using log K_oa_ as a predictive parameter is still somewhat similar to that of the equation in the corresponding parameter (lower left hand graph) in SI-Figure 5 (Tromp et al., 2019 – log K_sa_ = 0.778*log K_oa_ + 0.813 vs log K_sa_ = 0.807*log K_oa_ + 0.638), with less than 0.2 log units of difference between the slopes or intercepts.

### Lines of best fit for Log K_sa_ relationships

After modeling predictive relationships between physicochemical parameters and log K_sa_ estimates from this study, several refinements of the models were performed. In SI-Figure 6, all the lines of best fit from each modification are graphed against the log K_sa_ estimates from this study and Tromp et al., 2019 (gray circles). Surprisingly, there are only minor differences between most lines-of-best-fit. Using data only from this study (gray line), or using the full datasets without weighting (gray dashed line) are the two types of models that differer the most from the others. There are only slight changes in slope between higher confidence data (HCD). Specifically, in the cases of boiling points (both TEST and OPERA) and log K_oa_, differences between “Full” datasets weighted, and HCD datasets regardless of weighting are difficult to discern. While it is still recommended to use the HCD weighted dataset and model, it is encouraging that the slopes and intercepts are not changing dramatically between model refinements.


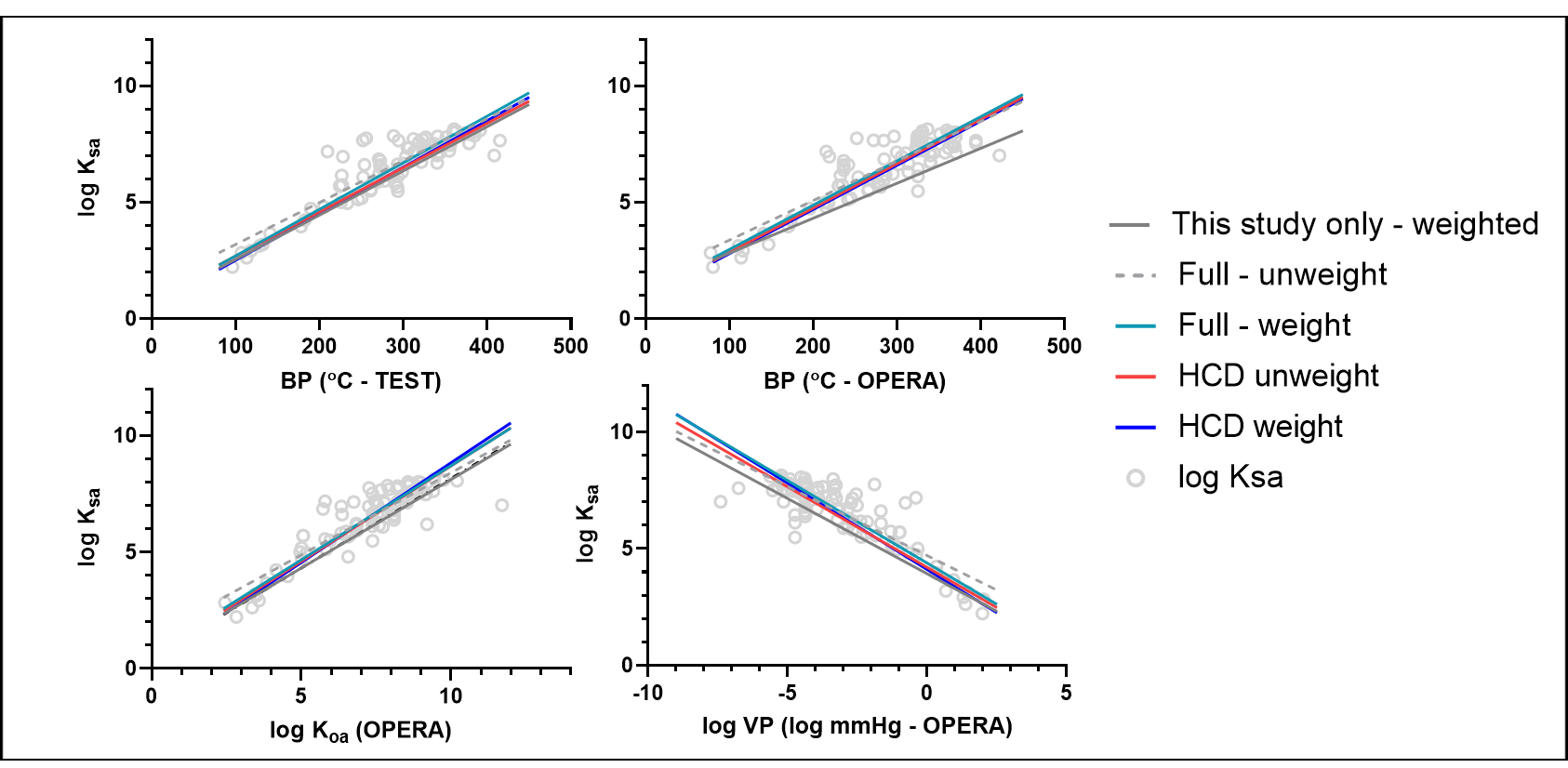


SI-Figure 6. Lines of best fit for linear regressions generated from this study or combined with another. Comparisons of lines of best fit include weighted and unweighed data. Gray circles are log K_sa_ values from this study and Tromp et al., 2019. HCD is high confidence data based models that have values with less than 1 log unit of SD.

## Model performance and comparisons

### Cross-validation

Repeated k-fold cross validation tests (10-repeated measures on 10-folds) were performed to evaluate the predictive performance between Full and HCD models of different chemical parameters (SI-Table 2). The outcomes of the cross validation (CV) are presented in SI-Table 2 including the averaged equation, R^2^ model fit of the fitted equation, the root mean squared error (RMSE), the mean absolute error (MAE), and the R-squared correlation between the observed outcome values and the predicted values by the model.


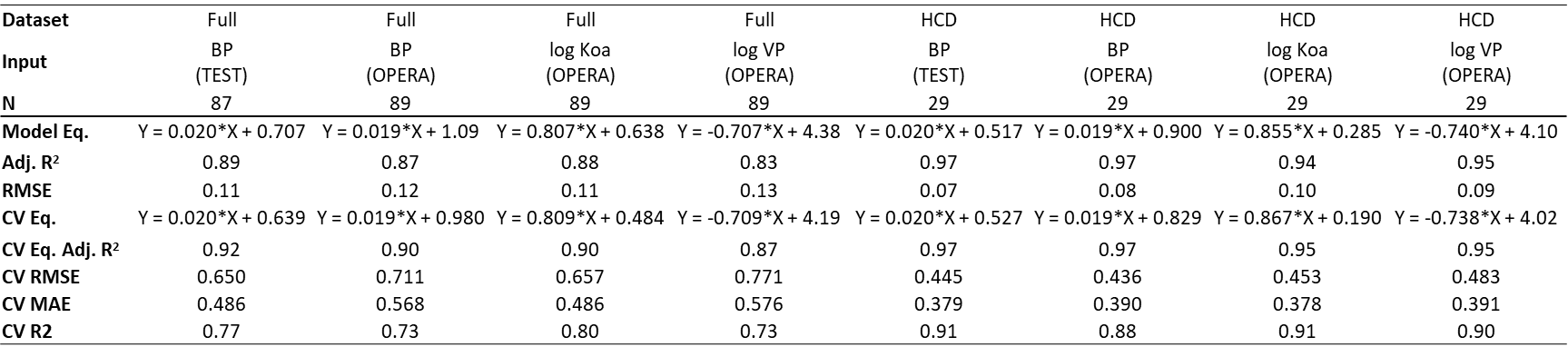
SI-Table 2. Table of linear regression performance and repeated k-fold cross validation performance for several combined data models using all (FULL) or high confidence data (HCD - less than 1 log unit SD).

Among the Full datasets, there are small differences among the CV outputs, with BP-TEST and log Koa OPERA performing the best, followed by BP-OPERA and log VP-based models. The min and max differences for RMSE and MAE among all 4 models is 0.121 and 0.090 log units, respectively. After refining the FULL dataset with higher confidence data (from 87-89 datapoints to 29), the HCD models also performed similarly amongst the different chemical inputs. The min and max differences for RMSE and MAE were 0.047 and 0.013, respectively among the HCD models. For both sets of data (Full and HCD), log VP was the worst performing model among the four, although well within 0.2 log units regardless of the performance measure. Models based on BP-Test data performed well amongst the other models regardless of dataset. Given the similarity in predictive performance, the cross validation exercise helped to provide estimates of error for each model, a slightly modified equation to use going forward, and the slight edge to BP or log K_oa_-based models over that of log VP.

## Example of air equivalency calculation

As a final demonstration, SI-Table 3 represents air concentration equivalents in ng/L that are calculated using equation 5 and converting k_e_ to R_s_ along with example deployment information and the HCD BP models described in this study for log K_sa_ and log k_e_ estimates.

From SI-Table 3, air concentration estimates between models vary in agreement among different compounds, although all are within an order of magnitude, with 13 out of 21 estimate pairs within 2-fold despite different models and inputs for K_sa_ and k_e_ values. Only 4 estimate pairs differ by over 3-fold, and all are SVOC compounds with estimates below 1 ng/L, or roughly parts per trillion levels.

This example dataset includes the compounds mentioned in Table 1 of the manuscript, but as previously noted, it is debatable whether reporting air equivalents is as relevant for SVOCs such as permethrin vs more volatile SVOCs and VOCs. Since the route of exposure for these less volatile compounds (ex: pyrene, permethrin, and chlorpyrifos) may not primarily lie in atmospheric uptake, in addition to having more uncertainty around air equivalency uptake, it is worth considering the relevancy in reporting air estimates for these compounds.

SI-Table 3. Air equivalency example calculation using HCD BP models for log K_sa_ and log k_e_ estimates

Finally, in SI-Figure 7, air equivalency values from SI-Table 3 were correlated together, and resulted in a Spearman’s r of 0.985 over 5 orders of magnitude. Deviations from a 1:1 relationship all occurred below 1 ng/L, or roughly a part-per-trillion with BP-TEST based values being higher than those derived from BP-OPERA.


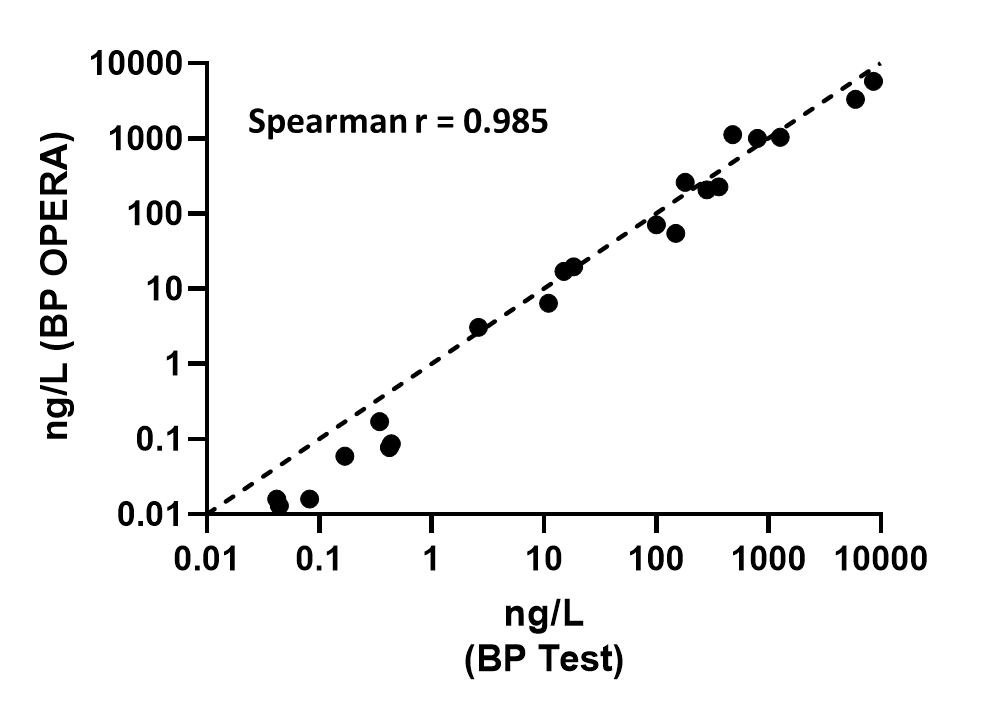


SI-Figure 7. Comparison of air equivalency estimates (ng/L) between two BP-based model sets (log Ksa (Eqs. 1 and 2) and log ke (Figure 3)) using example data and chemicals listed in Table 1. Dotted line represents a 1:1 relationship.

### Comparison of log K_sa_ values with other publications

From SI-Table 4, log K_sa_ estimates are given for each of the organic chemicals listed in Table 1 of the manuscript. Log K_sa_ models include all models and equations listed in SI-Table 2 apart from log VP models, as well as LFER-based estimates ^12^, and those from other publications using log K_oa_ based models ^4,13^. There are 9 models in total, ranging in R^2^ values from 0.33-0.99 using between 29-142 log K_sa_ estimates in each model (SI-Table 2). Notably, the Donald et al., 2019 model incorporated estimates for log K_sa_ in an outdoor setting, but temperatures still ranged approximately 20-25°C ^13^. Colored cells are used to identify low or high estimates among the nine models with blue or pink cells, respectively. Over half of the compounds in SI-Table 3 have log differences less than 1 (12 out of 22), and another 3 estimates differ by less than 2 log units. Not surprisingly, the largest discrepancies between models occur for SVOC compounds, and tend to be over 2 log units between the lowest and highest estimates. As compound volatility decreases, the relative importance of estimating atmospheric equivalency also decreases since exposures and uptake into the silicone are increasingly likely from other non-atmospheric routes of exposure. However, SI-Table 4 is encouraging considering the good agreement among all 9 models for VOC compounds excluding DCM. The lower bound estimates (DCM and ACO) are higher than other VOCs, and this could be from the transitory nature of very volatile compounds with higher rates of uptake than other VOCs, as well as limitations of some models that did not contain volatile compounds in the model data.


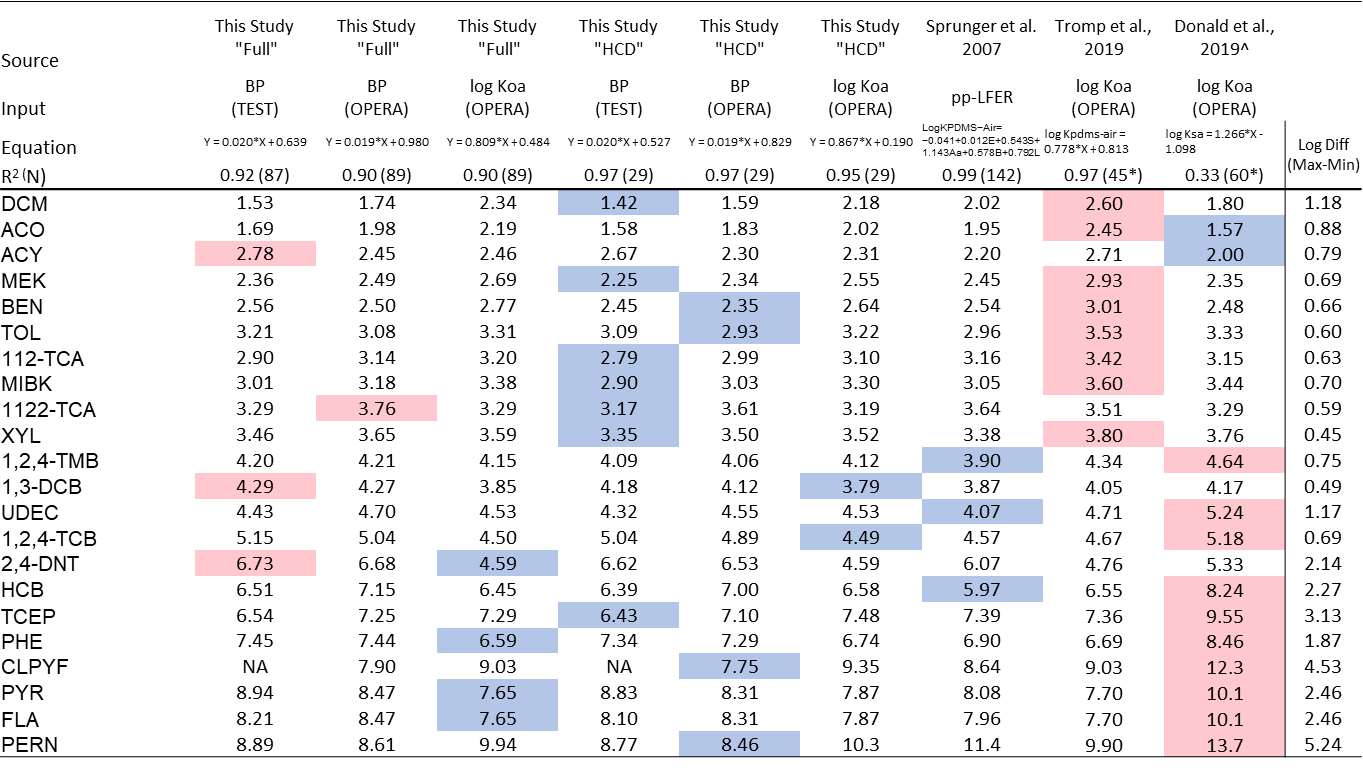
SI-Table 4. Log K_sa_ comparison among this study and other publications for VOC and SVOCs

Pink and blue colored cells refer to the highest and lowest log Ksa estimates among the 9 models, respectively.

SI-Figure 8. Comparison of models with R^2^ values ≤ 0.95 using compounds listed in Table 1 with models from Tromp et al., 2019 ^4^ and Sprunger et al., 2007 ^12^ do not differ dramatically for most of the compounds (less than 1 log unit). Compounds on the x-axis are listed from left to right with decreasing volatility. * indicates there was no BP (TEST) estimate for chlorpyrifos.

SI-Figure 8 represents some of most robust models from SI-Table 4. There is good agreement (less than 1 log unit) across model estimates that have R^2^ values of over 0.95 for log K_sa_ values across all compounds (15 out of 22 comparisons). This is promising when considering the range of chemistry, with extremes on either side of the volatility spectrum having the greatest differences between models (approximately 1 log unit or greater between lowest and highest estimate). Overall, this convergence around the most critical estimate of converting passive sampler extract data into atmospheric data (i.e. log K_sa_) helps this approach advance when estimating air concentrations from silicone extract data.

### Correlation of higher predictive HCD models with Sprunger et al., 2007 LFER estimates

A more thorough comparison with the silicone model from Sprunger et al., 2007 using LFER estimates than in SI-Table 3 can be made if using a larger chemical dataset. For this comparison, we used a list of internal chemicals identified after wearing SWBs for at least 3-days in a variety of applications. From this list of chemicals (281 compounds including VOCs and SVOCs), estimates of log K_sa_ were made using both Sprunger et al., 2007 and HCD models from this study. Those log K_sa_ estimates ranged from approximately 2-11 for HCD models, and 2-14 for LFER based models. LFER data was available for 190 out of 281 compounds ^14^, and estimates for BP and log K_oa_ were available for all compounds using EPA Comptox QSAR methodologies (TEST missing 13, OPERA missing 2 for boiling point, but not for the same compound resulting in at least 1 BP estimate for all 281 compounds and there were OPERA log K_oa_ estimates for all 281 compounds).

Spearman r correlation coefficients were made between log K_sa_ estimates for 185-190 compounds from BP and log Koa HCD models and Sprunger et al., 2007 (SI-Figure 9). Correlations were all over 0.9, with BP-TEST and Sprunger et al., 2007 log K_sa_ estimates correlating the best with an 0.97 value. A leveling off was seen in all three comparisons once log K_sa_ estimates were above approximately 8, indicating a lower ceiling of estimates for HCD models compared with Sprunger et al., 2007 estimates. Again, this discrepancy becomes less relevant when considering deviations on the higher end of the estimate range since these compounds are much more likely entering the SWB from other routes of exposure rather than air estimates. This finding promotes caution when reporting atmospheric equivalency values when log K_sa_ estimates are 8 or greater since the uncertainty around this estimate is likely larger regardless of the model used.


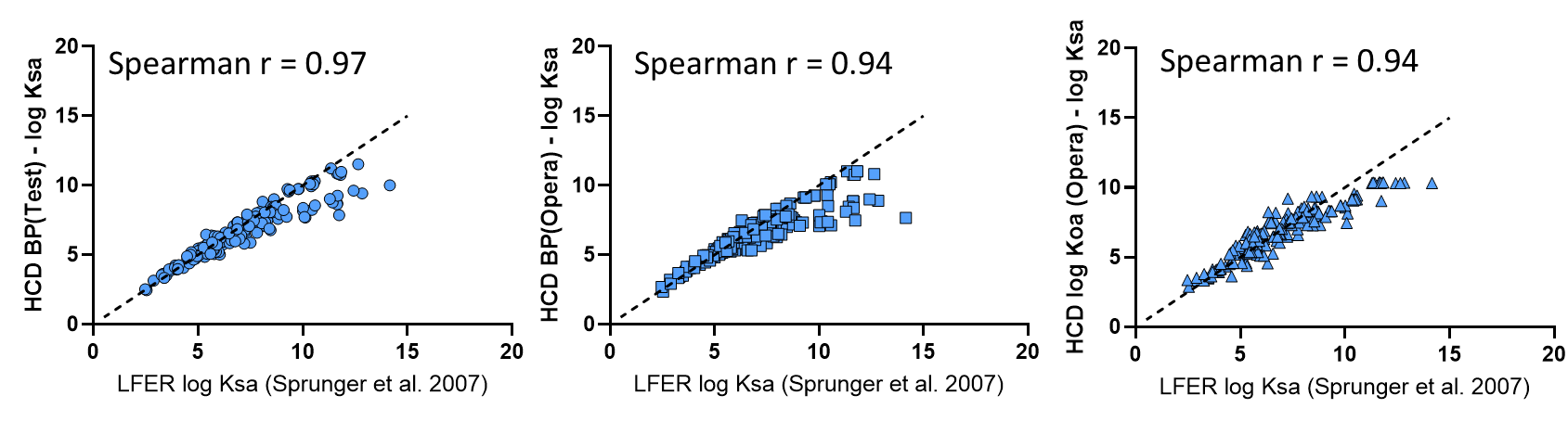


SI-Figure 9. Correlation between log Ksa estimates determined from HCD models and LFER-based model (Sprunger et al., 2007). Dotted line represents the 1:1 correlation line. Data includes 185-190 compounds that have been known to sequester to silicone wristbands (internal data).

## References

1 Huckins JN, Petty JD, Booij K. *Monitors of Organic Chemicals in the Environment*. 2006.

2 Bartkow ME, Booij K, Kennedy KE, Müller JF, Hawker DW. Passive air sampling theory for semivolatile organic compounds. *Chemosphere* 2005; **60**: 170–176.

3 Rusina TP, Smedes F, Koblizkova M, Klanova J. Calibration of Silicone Rubber Passive Samplers: Experimental and Modeled Relations between Sampling Rate and Compound Properties. *Environ Sci Technol* 2009. doi:10.1021/es900938r.

4 Tromp PC, Beeltje H, Okeme JO, Vermeulen R, Pronk A, Diamond ML. Calibration of polydimethylsiloxane and polyurethane foam passive air samplers for measuring semi volatile organic compounds using a novel exposure chamber design. *Chemosphere* 2019; **227**: 435–443.

5 Vrana B, Allan IJ, Greenwood R, Mills GA, Dominiak E, Svensson K *et al.* Passive sampling techniques for monitoring pollutants in water. *TrAC Trends Anal Chem* 2005; **24**: 845–868.

6 O’Connell SG, Kincl LD, Anderson KA. Silicone Wristbands as Personal Passive Samplers. *Environ Sci Technol* 2014; **48**: 3327–3335.

7 Donald CE, Scott RP, Blaustein KL, Halbleib ML, Sarr M, Jepson PC *et al.* Silicone wristbands detect individuals’ pesticide exposures in West Africa. *R Soc Open Sci* 2016; **3**: 160433.

8 Anderson KA, Points GL, Donald CE, Dixon HM, Scott RP, Wilson G *et al.* Preparation and performance features of wristband samplers and considerations for chemical exposure assessment. *J Expo Sci Environ Epidemiol* 2017. doi:10.1038/jes.2017.9.

9 Wang S, Romanak KA, Stubbings WA, Arrandale VH, Hendryx M, Diamond ML *et al.* Silicone wristbands integrate dermal and inhalation exposures to semi-volatile organic compounds (SVOCs). *Environ Int* 2019; **132**: 105104.

10 Aerts R, Joly L, Szternfeld P, Tsilikas K, De Cremer K, Castelain P *et al.* Silicone Wristband Passive Samplers Yield Highly Individualized Pesticide Residue Exposure Profiles. *Environ Sci Technol* 2018; **52**: 298–307.

11 Hammel SC, Phillips AL, Hoffman K, Stapleton HM. Evaluating the Use of Silicone Wristbands To Measure Personal Exposure to Brominated Flame Retardants. *Environ Sci Technol* 2018; **52**: 11875–11885.

12 Sprunger L, Proctor A, Acree Jr. WE, Abraham MH. Characterization of the sorption of gaseous and organic solutes onto polydimethyl siloxane solid-phase microextraction surfaces using the Abraham model. *J Chromatogr A* 2007; **1175**: 162–173.

13 Donald CE, Scott RP, Wilson G, Hoffman PD, Anderson KA. Artificial turf: chemical flux and development of silicone wristband partitioning coefficients. *Air Qual Atmosphere Health* 2019; **12**: 597–611.

14 Ulrich N; E S; Brown, TN; Watanabe, N; Bronner, G; Abraham, MH; Goss, KU. UFZ-LSER database v 3.2. Helmholtz Cent. Environ. Res.-UFZ. 2017.http://www.ufz.de/lserd (accessed 24 Mar2020).
